# Symptoms, Course, and Factors Related to Long-Term Morbidity, Including Differences between Infection Strains, in Patients with Long COVID in a Primary Care Clinic in Japan: An Observational Study

**DOI:** 10.3390/jcm13175019

**Published:** 2024-08-24

**Authors:** Kenji Baba, Seiko Kawai, Satoshi Iwase, Takahiro Ushida, Yasuhiro Tamura, Mariko Arimoto, Makiko Nojiri, Daisuke Watanabe, Nobutaro Ban

**Affiliations:** 1 Department of General Medicine, Medical Center, Aichi Medical University, 17-33 Kawagoe, Niki-cho, Okazaki 444-2148, Aichi, Japan; nobuban@aichi-med-u.ac.jp; 2 Center of Medical Education, Aichi Medical University School of Medicine, 1-1 Yazakokarimata, Nagakute 480-1195, Aichi, Japan; 3 Department of Neurology, Konan Kosei Hospital, 137 Omatsubara, Takaya-cho, Konan 483-8704, Aichi, Japan; 4 Department of Pain Relief Surgery & Multidisciplinary Pain Center, Aichi Medical University Hospital, 1-1 Yazakokarimata, Nagakute 480-1195, Aichi, Japan; 5 Department of Gastroenterology, Aichi Medical University School of Medicine, 1-1 Yazakokarimata, Nagakute 480-1195, Aichi, Japan; 6 Department of Otorhinolaryngology-Head and Neck Surgery, Aichi Medical University School of Medicine, 1-1 Yazakokarimata, Nagakute 480-1195, Aichi, Japan; 7 Department of Dermatology, Medical Center, Aichi Medical University, 17-33 Kawagoe, Niki-cho, Okazaki 444-2148, Aichi, Japan; 8 Department of Dermatology, Aichi Medical University School of Medicine, 1-1 Yazakokarimata, Nagakute 480-1195, Aichi, Japan

**Keywords:** long COVID, post-COVID condition, SARS-CoV-2, COVID-19

## Abstract

**Objectives:** The objectives were to investigate the clinical characteristics and course of long COVID, defined as the persistence of symptoms at least one month after the onset of COVID-19, in outpatients and to clarify differences in symptoms between SARS CoV-2 mutant strains. **Methods:** Our observational study in a primary care institution in Japan included 1053 patients with long COVID who visited our outpatient clinic between April 2021 and March 2023. Symptom distribution, performance status, and patient background at the time of the first outpatient visit were compared between infectious strains (Delta and before group and Omicron group). Background factors and symptoms related to time to remission were also analyzed. **Results:** The severity of COVID-19 in the acute phase was mild, moderate, and severe in 82.2%, 14.9%, and 2.9% in the Delta and before group; and in 97.6%, 1.7%, and 0.4% in the Omicron group, respectively. Vaccination coverage was significantly different between the Delta and before (37.1%) and Omicron groups (73.1%) (*p* < 0.001), probably due to the period of vaccine unavailability in the former group. Symptoms of fatigue and headache occurred most frequently, irrespective of infectious strain. The mean number of symptoms per patient was significantly higher in the Delta and before group than the Omicron group (3.4 vs. 2.7, *p* < 0.0001). The median time overall to remission of long COVID was 169 days. Cox hazard model analysis identified female sex, high body mass index, and dyspnea (but not infectious strain) as significant factors prolonging the time to remission (*p* < 0.05). **Conclusions:** Differences in the number of symptoms between infectious strains may be related to differences in viral virulence and/or vaccination coverage. However, the clinical course was found to be minimally influenced by the infectious strain. The present results should improve the understanding of prognosis in patients with long COVID from both the clinical and social perspectives.

## 1. Introduction

The spread of SARS-CoV-2, which began in Japan in early 2020, reached the eighth wave before gradually diminishing in March 2023. Accordingly, the Infectious Diseases Act in Japan relaxed the level of infection risk category after 8 May 2023. However, there has been a cumulative increase in the number of patients suffering from symptoms that persist or develop after COVID-19. There are two main clinical case definitions commonly in use for prolonged post-COVID symptoms, or “long COVID”, as published in the National Institute for Health and Care Excellence (NICE) guideline [1] and the WHO guideline [2].

Several large-scale studies have reported that long COVID can cause numerous symptoms throughout the body, that patients often complain of multiple symptoms, and that a significant proportion of patients complain of long COVID at more than one year after the initial infection [3,4,5]. As yet, there is no evidence-based treatment for long COVID [6].

Previous reports have suggested that the risk of developing long COVID decreases with repeated SARS-CoV-2 mutations, and that the distribution of long COVID symptoms differs depending on the mutant strain [3]. A recent study reported that the cumulative incidence of long COVID during the first year after infection caused by SARS-CoV-2 decreased along with the transition of the era of the infectious strain (difference between the Omicron and pre-Delta eras, –2.66 events per 100 persons; difference between the Omicron and Delta eras, –1.75 events per 100 persons) [7].

The risk of developing long COVID is reported to increase with the severity of acute COVID-19 [8]. It is also known that some patients with long COVID had only mild symptoms of COVID-19 [9]. However, there are insufficient data, especially from Japan, regarding the distribution of symptoms of long COVID in patients who had mild COVID-19 disease and on differences among the infectious strains.

In addition to the prolongation of various symptoms, long COVID is characterized by subsequent impairments of social and daily life, and the long-term failure of patients’ performance status (PS) has been identified as an important problem [6,10]. Meanwhile, an absence of understanding regarding the recovery time for long COVID has created challenges in social settings, such as the workplace, schools, and occasionally within families. The misunderstanding of their long-term suffering can place a great psychological burden on such patients.

In the present study, we examined the PS of patients with long COVID and the distribution of long COVID symptoms, as well as factors that lengthen the time until recovery to normal social and daily life. We also examined whether or not any of these parameters differed with respect to the SARS CoV-2 mutant strain. We believe that these findings will contribute to improving the clinical care of patients with long COVID and also enable a reduction in prejudice and misunderstanding toward those with long COVID.

## 2. Materials and Methods

### 2.1. Study Design and Setting

We conducted a retrospective, observational, no-control study at a single primary care institution in Japan and adhered to the Strengthening the Reporting of Observational Studies in Epidemiology (STROBE) cohort reporting guidelines [11].

### 2.2. Subjects

On 1 April 2021, our medical unit started treating patients with long COVID as defined by the NICE guideline; i.e., prolonged symptoms for at least 4 weeks after the onset of COVID-19. We studied 1053 patients with long COVID who had attended our medical unit by 31 March 2023. All of these patients had received treatment at another medical institution during the acute phase of COVID-19. In Japan, the Alpha strain was followed by the Delta strain, which was replaced by the Omicron strain from January 2022 [12,13]. As the period of dominance of the Omicron strain was reasonably clear, it was possible to identify the infectious strain with a fair degree of accuracy by the time of onset of COVID-19. However, the period of transition from the Alpha to Delta strains was less clear, and it was difficult to estimate the infectious strain based on the time of onset of the disease. Accordingly, patients infected by the end of December 2021 were termed the “Delta and before” group, and those infected on or after 1 January 2022 were termed the “Omicron” group. All recorded parameters were compared between the groups.

### 2.3. Clinical Parameters

Sex, age, the severity of COVID-19 at the time of the illness, vaccination status, body mass index (BMI), and the number of days between the COVID-19 diagnosis (confirmed positive PCR or antigen test) and the first visit were collected from all patients. Severity at the onset of COVID-19 was classified based on the Manual of Medical Treatment for COVID-19 of the Ministry of Health, Labour and Welfare of Japan [14]. Briefly, severity was graded according to oxygen saturation by pulse oximetry (SpO_2_) as “mild”, SpO_2_ > 96%; “moderate I”, 93% < SpO_2_ < 96%; “moderate II”, SpO_2_ < 93%, requiring oxygen administration; and “severe”, mechanical artificial respiration required for life support.

### 2.4. Symptoms

Using an open-ended question format, patients were asked at their first visit to “list all symptoms that are currently troubling them in their daily life and social activities and that they would like to get rid of”. They were also given the opportunity to add any additional relevant information regarding their symptoms. In each patient, we evaluated whether an existing symptom, irrespective of the cause, had worsened after the onset of COVID-19 to be considered a symptom of long COVID. We grouped the symptoms into 28 types (see Table S1) according to the typical symptoms reported in previous studies [3,4,5,15] and recorded the frequency of each symptom in the present patients according to strain group. The rate of each symptom was expressed as a percentage, calculated as the number of patients with that symptom divided by the total number of patients in the respective strain group.

To estimate the extent to which patients were able to perform social or domestic tasks, we assessed patients’ performance status (PS) at the time of their first visit using a PS classification system previously described for evaluating chronic fatigue syndrome [16] (see Table S2). In brief, a score of PS 7–9 indicates severe impairment of quality of life that makes it impossible to participate in society or carry out light duties at home, whereas a score of PS 1–2 indicates a normal social life in which work and housework are possible but symptoms are often felt, or the patients often need to rest due to their symptoms.

### 2.5. Assessment of Patients Who Achieved Remission of Symptoms

To confirm whether diseases other than long COVID were present, we performed clinical examinations according to patients’ symptoms in the first 4 weeks following the initial visit, and checked the results of any tests performed at other medical institutions just before visiting our institution. As a rule, all patients were then examined at monthly outpatient appointments, at which we asked the patients to confirm which symptoms remained, which were continuing to improve, and which had improved. We also provided guidance on how to live one’s daily life, including pacing therapy for patients with fatigue and treatments for symptoms. We assessed whether they had achieved symptom remission after treatment. We defined remission as: (1) complete disappearance of symptoms or regaining of a normal social life even if symptoms remained at the level of PS 0–2 in patients who had scored PS 3 or higher, and (2) complete disappearance of symptoms (improvement to PS 0) in patients who had scored PS 1–2. The date at which remission was confirmed at a regular follow-up visit was taken as the date of remission. We calculated the distribution of days from the date of COVID-19 diagnosis to remission and performed analysis of various parameters, including infectious strain type, to identify the factors affecting the time to remission.

### 2.6. Statistical Analysis

All values are presented as the median values (min–max), except for the rates of symptoms (%). The Mann–Whitney U test or χ^2^ test was used to compare the parameters between the groups. The Cox proportion hazard model was used to clarify the factors related to the length of time to remission. *p* < 0.05 was considered to indicate a significant difference. All statistical analyses were performed using Bell Curve (ver. 4.06) or StatView (ver. 5.0) software.

### 2.7. Ethics

As this observational study used only medical information and patient background information, patient consent was obtained via the opt-out method on our institution’s website, which provided information about the purpose and design of the study and explained the consequences of refusing to consent. This study was approved by the Ethical Review Committee of Aichi Medical University (approval No. 2024-027).

## 3. Results

### 3.1. Patients

Table 1 lists the patients’ demographic and clinical characteristics. There were 498 patients in the Delta and before group and 555 in the Omicron group. There were no significant differences in sex ratio, age distribution, or BMI between the strain groups. There was a higher proportion of women in both groups. In terms of the severity of illness at the onset of COVID-19, symptoms were significantly more severe in the Delta and before group than the Omicron group (*p* < 0.001). Regarding vaccination coverage before the onset of long COVID, the vaccination rate was significantly lower (*p* < 0.001) in the Delta and before group than in the Omicron group. Regarding the distribution of the PS at the first visit, significantly more patients in the Omicron group than the Delta and before group had a PS in the range 7–9.

**Figure 1:**
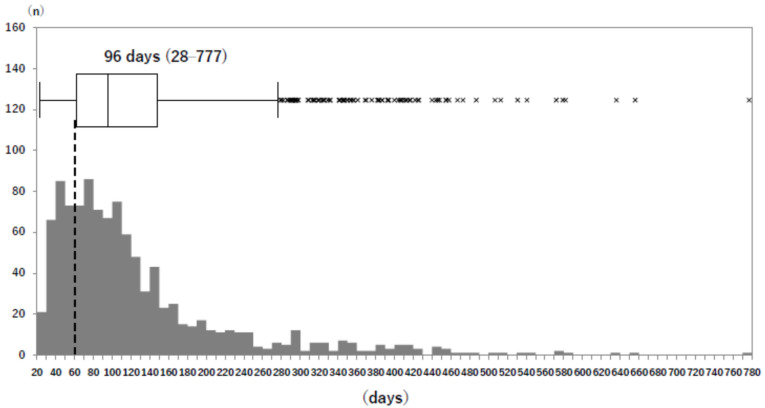
Overall distribution of days from COVID-19 diagnosis to first long COVID outpatient visit. The dotted line indicates the 60 days in the WHO clinical case definition of post-COVID condition. Box-and-whisker plots are shown with a 25-percentile base, a median and a 75-percentile top.

The median times from the time of COVID-19 illness to the long COVID outpatient visit was 96 days in the overall patients, 101 days in the Delta and before group, and 95 days in the Omicron group (no significant difference between the infection strain groups). Two hundred and forty-seven patients (23.5%) visited the outpatient clinic with a duration of symptoms of less than the 2 months (60 days), as stated in the WHO definition [2]; i.e., symptoms persisting for at least two months after the onset of COVID-19 that cannot be explained by an alternative diagnosis (Figure 1).

### 3.2. Long COVID Symptom Distribution

**Figure 2:**
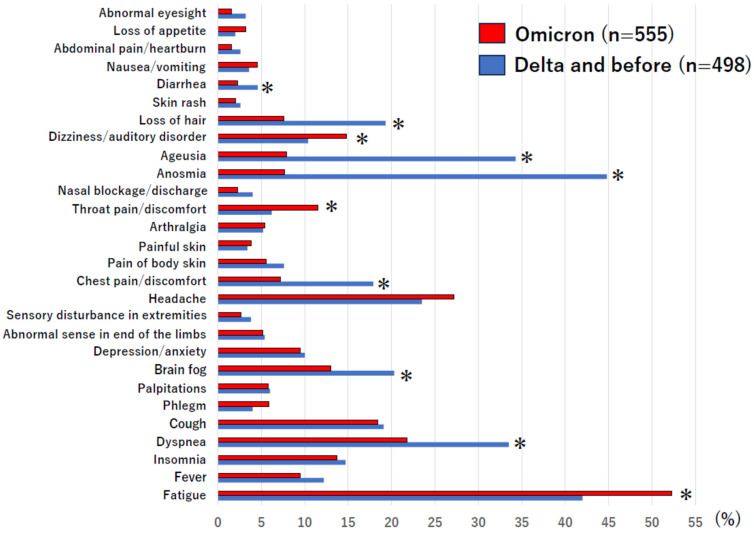
Distribution of symptoms of long COVID mentioned at the first outpatient visit, expressed as a percentage of the total number in each group for each of the Delta and before group and the Omicron group. * Denotes a significant difference (χ^2^ test).

Figure 2 shows the distribution of long COVID symptoms according to strain group. The rates of anosmia, ageusia, brain fog, loss of hair, dyspnea, diarrhea, and chest pain/discomfort were all higher in the Delta and before group than in the Omicron group, whereas the rates of fatigue, dizziness/auditory disorder and throat pain/discomfort were higher in the Omicron group than in the Delta and before group. The following features were common to both groups: (1) fatigue, which was the most frequent complaint (>40%), and (2) pain symptoms, such as headache, chest pain, body pain, musculoskeletal pain, and pharyngeal pain, which were reported by nearly half of the patients (Delta and before group, 46.6%; Omicron group, 48.5%).

The mean number of symptoms reported per person for the symptoms classified in this study was 3.7 (median of 3 symptoms; range of 1–11) in the Delta and before group and 2.7 (median of 2 symptoms; range of 1–13) (significant difference, *p* < 0.0001).

We examined the distribution of symptoms for each group according to whether or not ≥180 days had passed after the diagnosis of COVID-19. In the Delta and before group, there was no significant difference in the number of symptoms per person regardless of the number of days (<180 days: mean of 3.7, median of 3 (1–10); ≥180 days: mean of 3.5, median of 3 (1–11)). In contrast, in the Omicron group, the patients had significantly fewer symptoms per patient when the first visit was ≥180 days compared to <180 days (<180 days: mean of 2.8, median of 3 (1–13); ≥180 days: mean of 2.2, median of 2 (1–6)). For each symptom, the rate of fatigue in the Omicron group and cough symptoms in the Delta and before group were significantly lower at ≥180 days than at <180 days. The rate of muscle pain was higher at ≥180 days in the Delta and before group (Figure S1).

**Figure 3:**
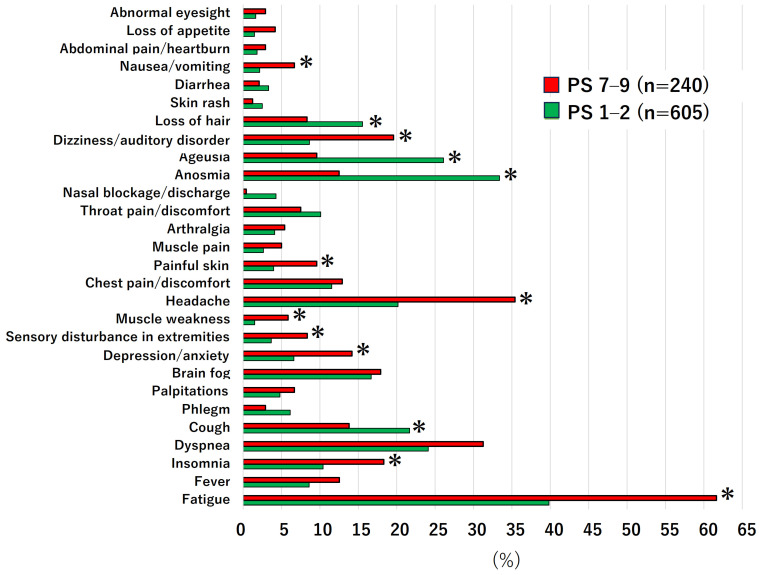
Distribution of symptoms at the first visit according to good (1–2) and poor (7–9) performance status (PS) (see Table S2) at the first visit among all patients. Values are expressed as the percentage per total number of patients in each PS group. * Denotes a significant difference (χ^2^ test).

We compared the distribution of symptoms between the patients with a good PS (PS 1–2, n = 605) and a poor PS (PS 7–9, n = 240) (Figure 3). In the poor PS group, fatigue, headache, dizziness/auditory disorder, insomnia, and nausea/vomiting were the most frequent symptoms and significantly more frequent than in the good PS group. In the good PS group, the most frequent symptoms, in descending order, were fatigue, anosmia, ageusia, and dyspnea. The frequencies of anosmia and ageusia in particular were significantly higher in the good PS group (PS 1–2). The rates of dyspnea and brain fog were relatively high in both groups and did not show a significant difference between the PS groups.

### 3.3. Outcomes in the Long COVID Patients Who Visited Our Clinic

As of 31 March 2024 (12 months after the last enrolment of long COVID patients on 31 March 2023), we have evaluated the outcomes in all 1053 patients. Of these, 371 patients stopped coming to the clinic in the first 4 weeks following the initial visit, and 355 stopped coming during the course of treatment. The time from COVID-19 diagnosis to dropout in these 355 patients was 177 days (28–1055). In contrast, 295 patients met the criteria for symptom remission. Thirty-two patients were still undergoing treatment. The number of days of treatment since the diagnosis of COVID-19 in these patients was 641 days (398–1477) (see Figure S2).

### 3.4. Analysis of Patients Who Achieved Remission of Symptoms

**Figure 4:**
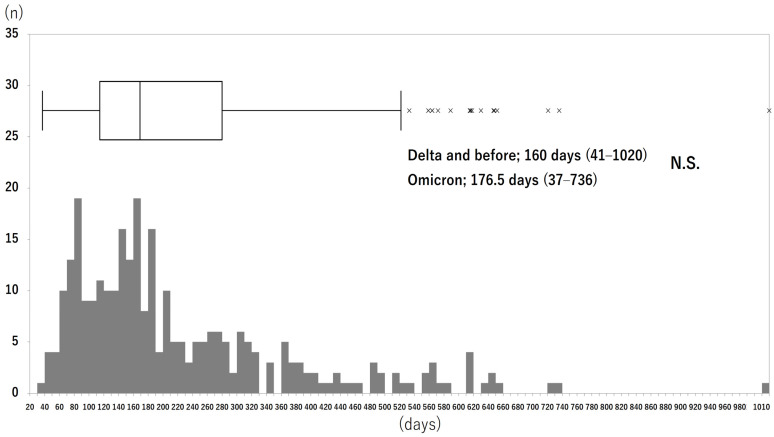
Distribution of the number of days from COVID-19 diagnosis to the date of confirmed remission in patients with remission (see text for definition), irrespective of the infectious strain. N.S.; not significant. Box-and-whisker plots are shown with a 25-percentile base, a median and a 75-percentile top.

In the patients who achieved remission, 34.2% were in the Delta and before group and 65.8% were in the Omicron group. Figure 4 shows the distribution of the number of days from the COVID-19 diagnosis to the date of confirmation of remission. The rate of patients achieving complete remission (from PS1–2 to PS0) was 58.6%, and that of the other remission criteria (from PS3 or higher to PS1–2) was 41.4% overall. The ratio of complete remission vs. that of the other remission criteria was not significantly different between the infectious strain groups; 66% vs. 34% in the Delta and before group and 55% vs. 45% in the Omicron group (*p* = 0.091, χ^2^ test).

The median number of days to remission was 169 days (37–1020) overall, 160 days (41–1020) in the Delta and before group, and 176.5 days (37–736) in the Omicron group (no significant difference between the infection strain groups) (*p* = 0.3334, Mann–Whitney U test).

There was no significant difference between the infectious strains in terms of the background parameters of the male/female ratio, age distribution, and period from the time of COVID-19 illness to the first outpatient visit: the male/female ratio was 47%/53% in the Delta and before group and 39%/61% in the Omicron group (*p* = 0.1798, χ^2^ test); the age distribution was 42 years (14–78) in the Delta and before group and 42 years (14–89) in the Omicron group (*p* = 0.4799, Mann–Whitney U test); and the period until the first visit was 70 days (27–582) in the Delta and before group and 87 days (23–382) in the Omicron group (*p* = 0.1114, Mann–Whitney U test).

In the analysis of factors affecting the length of time to remission, the patient backgrounds and symptoms for which the distribution rates were ≥10% in either the Delta and before or Omicron groups were selected. The results show that the female sex, higher BMI, and dyspnea were associated with a longer time to remission (*p* < 0.05, Cox proportion hazard analysis), but there was no effect of the infectious strain type. In contrast, cough and insomnia appeared to be related to a shorter duration until remission. Differences in the infectious strains, other symptoms, and backgrounds, including the vaccination status and degree of PS were not significantly associated with prolonged illness (Table 2).

## 4. Discussion

There are four major findings of the present study. (1) A total of 247 patients (23.5%) visited our primary care clinic with a shorter duration of symptoms after COVID-19 diagnosis than that set by the WHO for a case definition of long COVID; (2) the distribution of long COVID symptoms varied according to the infectious strain; (3) at >180 days from the onset of COVID-19, the number of symptoms and the rate of fatigue per patient had significantly decreased in the Omicron group but not in the Delta and before group; and (4) half of the patients required more than 169 days to return to their normal social life, with factors of the female sex, obesity, and dyspnea (but not the type of infectious strain), prolonging the time to remission.

The unique features of the present study are as follows: (1) PS was applied for the clinical evaluation of all patients with long COVID; (2) it focused not on the duration of symptoms of long COVID but on the time taken for patients with PS3 or more to return to normal social and daily life; and (3) it analyzed the factors involved in the prolongation of the period until remission for each infectious strain.

To the best of our knowledge, there is no clear indicator of the severity of long COVID. Fatigue is the most frequent symptom among those with long COVID and has a strong impact on social life, as shown in Figure 3. Furthermore, the main concern of long COVID patients is when their symptoms will subside and when they will be able to lead a normal social life. For this reason, the main focus in the present study was the ability to lead a normal social life, and we consider that the evaluation of PS using a classification framework previously used for chronic fatigue syndrome [16] was also meaningful for evaluating various symptoms in long COVID. Although the validation of PS for this purpose is an important focus of future research toward establishing a universal indicator for long COVID, we consider that this perspective offered by the present study can lead to the provision of more realistic information to patients with long COVID.

Of the 247 patients who visited the outpatient clinic for investigation of sequelae within 2 months after COVID-19 infection, 185 patients met the WHO clinical criteria for post-COVID condition, which suggests that there is a high need for medical care before reaching the symptom duration defined by the WHO. Although no evidence-based treatment for long COVID has been established, it would not be inappropriate to guide patients toward early interventions. In this context, the WHO definition of post-COVID condition may warrant reconsideration.

The severity of acute COVID-19 caused by the Omicron strain is known to be considerably less than those that preceded it (i.e., the Delta strains), probably due to differences in virulence among the infectious strains [3], which accounts for the different backgrounds of severity of acute COVID-19 between the present strain groups. It is noteworthy that the majority of long COVID patients seen at our primary care clinic had mild COVID-19, regardless of the infectious strain. All of these patients had received treatment for COVID-19 at another medical institution. It is likely that patients with moderate or severe symptoms who had been hospitalized would have received follow-up after discharge at the outpatient department of that same hospital. This is probably why the majority of patients with long COVID who came to our clinic were only mildly ill in the acute phase of COVID-19. A systematic review of cases of long COVID after mild COVID-19 showed that fatigue was the most common symptom and that symptoms, including fatigue, dyspnea, headache, and chest pain, affected social activity [8], which is consistent with the present results.

Regarding differences in long COVID symptoms between infectious strains, it has been suggested that fatigue and myalgia were more frequent in the Omicron strain than the Delta strain, whereas the incidence of sleep disturbances was lowest in Delta compared to other infectious strains [17]. Furthermore, Magnusson et al. [18] reported that the prevalence of long COVID symptoms was lower in Omicron than Delta strains at more than three months after COVID-19 infection. The results of the present study are in agreement with these reports in terms of fatigue and the number of symptoms, but not regarding the incidence of myalgia and sleep disturbances. These discrepancies may be due to various factors, such as the method of data extraction and racial differences, and indicate that further accumulation of data is still necessary. Nevertheless, it is not difficult to imagine that the present findings of lower distributions of long COVID symptoms and a lower number of symptoms per patient in the Omicron group than in the Delta and before group might be related to differences in virulence between the strains, such as differences in symptom distribution at the time of acute COVID-19 [6,19]. Furthermore, the present results appear to be due to the higher rate of vaccination coverage in the Omicron group compared to the Delta and before group. In Japan, vaccination is systematized by the government, and because the vaccination program started during the Delta strain epidemic, those who had been infected before then did not receive vaccination. We consider that this is the reason for the lower vaccination rate in the Delta and before group than in the Omicron group. However, recent systematic reviews [20,21] have shown that the impact of vaccines on the development of long COVID is not always clear, partly due to problems with research methods. This also remains an important research question.

In contrast, the present results of higher rates of fatigue and poor PS in the Omicron group than in the Delta and before group (Figure 2 and Table 1) suggest that from the perspective of social activity, long COVID is more serious in the Omicron group than in other strain groups. In other words, there might be a heightened need for social understanding and support systems, right from the early stage, in patients with long COVID in this strain group.

The analysis of factors affecting prolonged days to remission identified the female sex, higher BMI, and dyspnea as contributing factors (Table 2). It is known that the proportion of female patients with long COVID is higher also in Japan [15], but it is interesting to consider other factors such as unwanted autoimmunity after infection with COVID-19 [6], which might be more common in women, as is the case in connective tissue diseases. This is likely to be an important research theme. An observational study of healthcare workers affected by COVID-19 reported a significantly longer time to long COVID recovery when BMI was >25 kg/m^2^ [22]. Obesity is known to be a risk for COVID-19 severity [23], and it has been shown that genetically engineered obese mice are more likely to develop cytokine storms during the extreme phase of COVID-19 infection compared to wild-type mice [24]. The hypothesis that the immune response in obese patients may be responsible for the prolonged time to long COVID remissions is an interesting topic for future research. Dyspnea is another common prolongation factor that is known to be related to various factors other than oxygen supply problems, including muscle weakness, functional imbalance of skeletal muscles working during inspiration and expiration, fatigue, and/or psychogenic factors. These complex factors might be responsible for the prolongation of time to remission.

Regarding the time course until remission, our results suggest that once a patient has long COVID, even by the Omicron strain, the time course to remission may be the same as that of long COVID caused by the Delta strain. Xie et al. reported that the cumulative incidence of long COVID may decrease with mutations, but the risk of long COVID remained substantial even among vaccinated persons who had SARS-CoV-2 infection in the Omicron era [7]. These indicate that even by the Omicron strain, we must never be naïve about long COVID.

It is noteworthy that at least 32 of the present patients have not fully recovered and could require long-term treatment (Figure S2). Furthermore, half of these patients continue to complain of fatigue, raising the issue of its relationship with myalgic encephalomyelitis/chronic fatigue syndrome, in which fatigue is considered similar to that in long COVID [6,25]. The factors affecting such patients, including concomitant diseases, are also an important question of interest and currently under investigation.

According to the fear avoidance model in chronic pain proposed by Vlaeyen et al. [26], psychological factors, such as insomnia, anxiety, and fear, combined with family and social stress from destructive thoughts about symptoms (negative thoughts and stigma from surroundings), lead to behavioral patterns of avoidance; i.e., withdrawal, inactivity, and worsening depression, resulting in a vicious cycle that worsens the symptoms. This model might also apply to long COVID, and the vicious cycle of fear avoidance and secondary psychiatric problems with long COVID should be avoided as much as possible. In this context, the finding of a median time to remission of 169 days is also considered meaningful in terms of the concerns of long COVID patients, who do not know what the future holds.

In patients who have a risk of prolongation until the remission of long COVID, helping them to understand this risk and exercise patience in their lives could reduce the likelihood of them falling into a secondary depressive state. Furthermore, informing workplaces of the prolongation risk and promoting understanding might lead to the provision of patient-specific social support until remission. In contrast, the factors of cough and insomnia appear to be associated with a shorter duration until remission. As far as we know, there are few reports showing that cough or insomnia in long COVID tends to subside in a shorter period of time than other symptoms. This might be due to the fact that most of the patients in the present study had mild COVID-19 in the acute phase. Further investigation is needed regarding this finding.

In the present study, according to the definition of long COVID, various tests related to clinical symptoms (e.g., tests for dyspnea, chest CT, pulmonary function tests, cardiac physiology tests) were performed to confirm the absence of significant abnormalities. Therefore, we consider the association between long COVID symptoms and the usual laboratory tests to be low. However, the search for biomarkers related to symptoms and clinical course is an important topic for future research that is currently being planned.

The relationship between treatment and the course of long COVID was not examined in this study for the reason that long COVID was originally treated symptomatically and not according to a fixed protocol. Until now, the potential utility of several drugs has been reported for the treatment of long COVID [6]. Studying the effects of such drugs would enable investigation of their potential impact on the disease picture in the future.

There are limitations in the present study. This observational study evaluated patients who visited a single-center "post-COVID outpatient clinic", without a control group. Accordingly, the outcomes for each recognized variant could not be evaluated independently. The accuracy of the background infectious strain is inadequate, as this was determined by the time of onset of COVID-19, before the patients visited our clinic. It is well known that the overall mortality differed greatly between the Delta strain and the strains that followed, resulting in a marked difference in the number of survivors. In contrast, patients with milder symptoms of long COVID may have chosen not to visit the clinic. From this perspective, the present study has a selection bias. We did not establish patients’ backgrounds regarding concomitant diseases. The long COVID patients in this study were not treated in a uniform manner according to their symptoms. A significant number of patients stopped coming to the clinic during treatment, limiting the number of patients for whom the final outcome could be ascertained. Therefore, there are also limitations in the present analysis of factors, particularly in relation to remission.

## 5. Conclusions

We conducted an observational study of 1053 patients with long COVID at a primary care institution in Japan. In approximately one quarter of all patients, most were mildly ill in the acute phase of COVID-19 and required medical care earlier than the 2 months set by the WHO for a case definition of long COVID. The distribution of long COVID symptoms varied according to the infectious strain, consistent with the findings of previous studies. Our findings suggest the possible suitability of performance status for evaluating daily and/or social activities in patients with long COVID. Furthermore, we found that half of the patients required more than 169 days to return to their normal social life, with the factors of obesity and dyspnea prolonging the time to remission, but the clinical course was found to be less affected by the infectious strain. These results indicate that long COVID should still be considered a serious issue even if the infectious strain mutates. We believe that the present results will be useful for advising patients who are worried about the future and will provide information such as the clinical course and the risk of prolonged illness at the primary care level, thus enabling a reduction in prejudice and misunderstanding toward patients with long COVID.

## Figures and Tables

**Table 1:** Demographic and clinical characteristics.

	Delta and Before (n = 498)	Omicron (*n* = 555)	
Male/Female (%)	47.4/53.6	47.3/53.7	N.S. *
Age (years)	41.9 (15–87)	41.0 (15–89)	N.S. *
BMI (kg/m^2^)	22.2 (14.9–54.4)	21.9 (14.1–45.8)	N.S. *
Period (days) until visiting long COVID outpatient clinic after onset of COVID-19 **	101 (28–777)	95 (28–448)	N.S. *
Severity of COVID-19: mild/moderate I/moderate II/severe (%)	82.2/6.6/8.3/2.9 (*n* = 484)	97.6/0.7/1.3/0.4 (*n* = 536)	*p* < 0.001 **
Vaccination Y/N (%)	37.1/67.9 (*n* = 467)	73.1/26.9 (*n* = 554)	*p* < 0.001 **
Performance status: 1/2/3/4/5/6/7/8/9 (%)	38.1/30.5/5.8/4.7/1.6/ 2.7/12.8/3.5/0.4 (*n* = 485)	16.9/34.1/5.2/4.5/6.2/ 3.0/22.7/6.4/1.1 (*n* = 534)	*p* < 0.001 **

BMI, body mass index; N.S., not significant. * Chi-squared test ** Mann–Whitney U test.

**Table 2:** Analysis of factors affecting the time to remission.

Covariate	Level	Hazard Ratio (95%CI)	
Infectious strain	Omicron/Delta and before	0.875 (0.623–1.228)	N.S.
Sex	F/M	0.728 (0.542–0.977)	*p* = 0.0341
Age	Years	0.998 (0.989–1.008)	N.S.
Vaccination	Y/N	1.095 (0.815–1.471)	N.S.
BMI		0.959 (0.929–0.989)	*p* = 0.0072
Performance status		1.037 (0.978–1.099)	N.S.
Fatigue	Y/N	0.951 (0.716–1.262)	N.S.
Fever	Y/N	0.867 (0.570–1.318)	N.S.
Insomnia	Y/N	1.557 (1.048–2.313)	*p* = 0.0284
Dyspnea	Y/N	0.741 (0.552–0.996)	*p* = 0.0468
Cough	Y/N	2.295 (1.682–3.132)	*p* < 0.001
Brain fog	Y/N	0.780 (0.542–1.123)	N.S.
Depression/anxiety	Y/N	0.962 (0.615–1.506)	N.S.
Headache	Y/N	0.814 (0.576–1.150)	N.S.
Chest pain/discomfort	Y/N	0.861 (0.578–1.284)	N.S.
Throat pain/discomfort	Y/N	1.209 (0.797–1.833)	N.S.
Anosmia	Y/N	1.184 (0.772–1.817)	N.S.
Ageusia	Y/N	0.826 (0.613–1.327)	N.S.
Dizziness/auditory disorder	Y/N	1.099 (0.698–1.729)	N.S.
Loss of hair	Y/N	1.103 (0.674–1.804)	N.S.

Cox proportional hazard analysis. Symptoms for which the distribution rates were ≥10% in either the Delta and before or Omicron groups were selected for analysis of the prolongation factors. In analysis of hazard for the event of remission, a hazard ratio significantly lower than 1 indicates that the event is unlikely to occur (i.e., the symptoms are likely to persist). Conversely, a hazard ratio greater than 1 indicates the opposite. BMI, body mass index; CI, confidence interval; N.S., not significant.

## Data Availability

The original contributions presented in this study are included in the article/Supplementary Materials. Further inquiries can be directed to the corresponding author.

## Supplementary Materials

The following supporting information can be downloaded at: https://www.mdpi.com/article/10.3390/jcm13175019/s1, Figure S1: The distribution of symptoms for each infection strain group for the number of days from the time of COVID-19 diagnosis to the date of first long COVID visit of <180 days and of ≥180 days. Values are expressed as the percentage per total number of cases for each time frame. * Denotes a significant difference (χ^2^ test). Figure S2: Outcomes in the 1053 patients who visited our post-COVID outpatient clinic. Table S1: Classification of symptoms. Table S2: Performance status of long COVID patients.
