# Supplementary material for: Symptoms, Course, and Factors Related to Long-Term Morbidity, Including Differences between Infection Strains, in Patients with Long COVID in a Primary Care Clinic in Japan: An Observational Study
Source: J Clin Med. 2024 Aug 24;13(17):5019. doi: 10.3390/jcm13175019 (PMC11396328; doi:10.3390/jcm13175019)

Table S1: Classification of symptoms

Systemic

Fatigue	Fever	Insomnia

Respiratory & Cardiac

Dyspnea	Cough	Phlegm	Palpitations

Neurologic & Psychiatric

Brain fog*	Depression/anxiety**	Abnormal sense in end of the limbs	Muscle weakness

Pain

Headache	Chest pain/discomfort	Painful skin	Muscle pain	Arthralgia

Otorhinolaryngologic

Throat pain/discomfort	Nasal blockage/discharge	Anosmia	Ageusia	Dizziness/auditory disorder

Dermatologic

Loss of hair	Skin rash

Gastrointestinal

Diarrhea	Nausea/vomiting	Abdominal pain/heartburn	Loss of appetite

Optic

Abnormal eyesight

* Brain fog includes cognitive impairment, dull head and poor powers of concentration.

** Depression/anxiety includes impairment of sexual activity.

Table S2: Performance status of Long COVID patients

0	Able to carry out normal daily social activities without symptoms
1	Able to carry out normal daily social activities and work, but often feels symptoms
2	Able to carry out normal daily social activities and work, but often needs a rest because of symptoms
3	Several days a month, unable to carry out normal daily social activities and work because of symptoms
4	Several days a week, unable to carry out normal daily social activities and work because of symptoms
5	Unable to carry out normal daily social activities and work. Able to carry out light duties, but several days a week needs a rest at home
6	Able to carry out light duties only on good days, but after more than half a week needs a rest at home
7	Able to take care of oneself without assistance, but unable to carry out normal daily social activities or light work duties
8	Able to take care of oneself with assistance, but must be in bed more than half a day
9	Unable to take care of oneself, needs daily assistance, and must be in bed all day

Figure S1

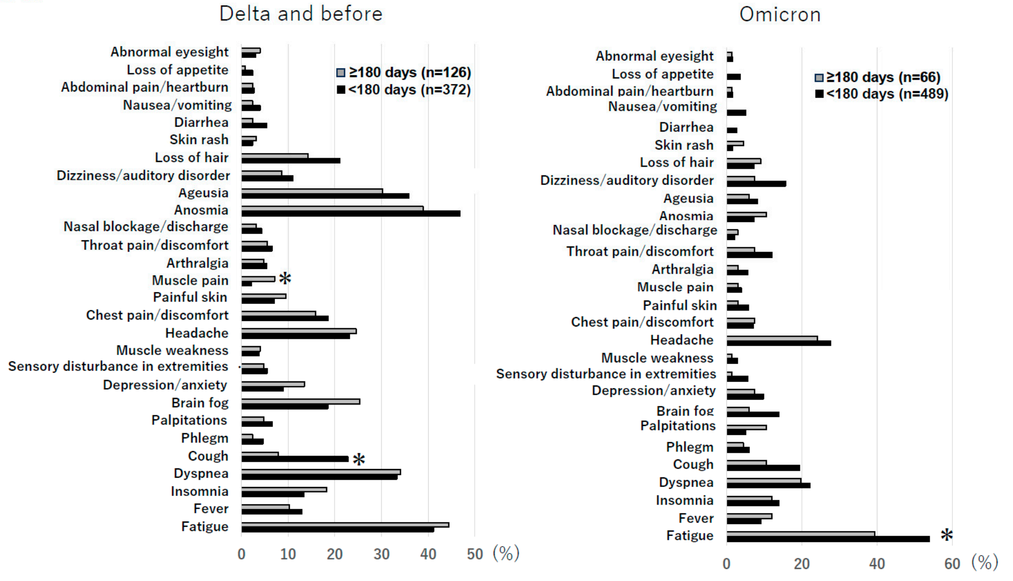

Figure S2

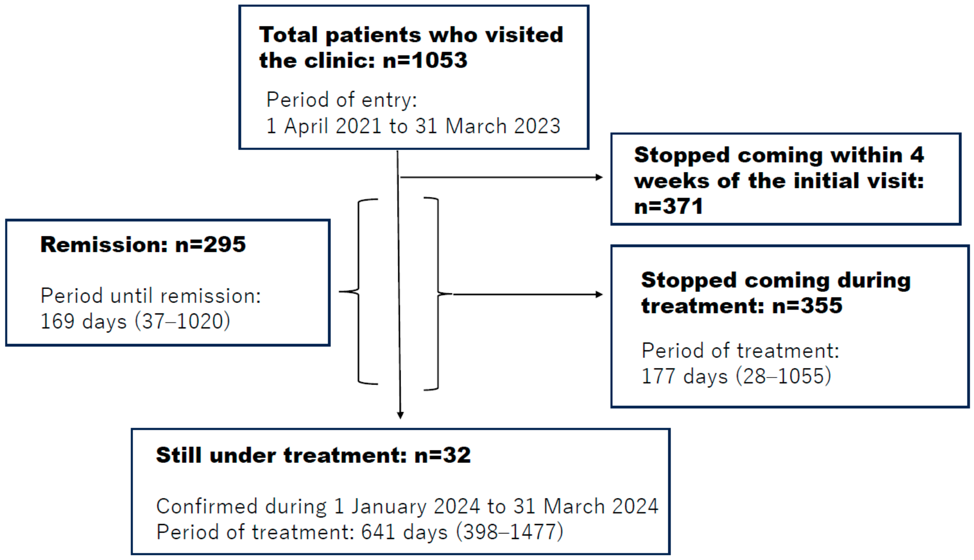

Supplement: Supplementary file 1 [file jcm-13-05019-s001.zip › jcm-3153451-supplementary.pdf]
